# The Report of Access and Engagement With Digital Health Interventions Among Children and Young People: Systematic Review

**DOI:** 10.2196/44199

**Published:** 2024-01-17

**Authors:** Lisa Whitehead, Suzanne Robinson, Diana Arabiat, Mark Jenkins, Evalotte Morelius

**Affiliations:** 1 School of Nursing and Midwifery, Edith Cowan University Joondalup Australia; 2 Centre for Postgraduate Nursing Studies, University of Otago Christchurch New Zealand; 3 The Centre for Evidence Informed Nursing, Midwifery and Healthcare Practice Joondalup Australia; 4 Australian Research Council Centre of Excellence for the Digital Child Joondalup Australia; 5 Maternal and Child Nursing Department, Faculty of Nursing, The University of Jordan Amman Jordan; 6 Department of Health, Medicine and Caring Sciences, Linköping University Linköping Sweden

**Keywords:** access, engagement, digital health technology, mobile phone, children

## Abstract

**Background:**

Digital health interventions are increasingly used to deliver health-related interventions for children and young people to change health behaviors and improve health outcomes. Digital health interventions have the potential to enhance access to and engagement with children and young people; however, they may also increase the divide between those who can access technology and are supported to engage and those who are not. This review included studies that reported on the access to or engagement with digital health interventions among children and young people.

**Objective:**

This review aims to identify and report on access and engagement in studies involving digital health interventions among children and young people.

**Methods:**

A systematic review following the Joanna Briggs Institute methods for conducting systematic reviews was conducted. An electronic literature search was conducted for all studies published between January 1, 2010, and August 2022, across sources, including MEDLINE, CINAHL, and PsycINFO. Studies were included if they examined any aspect of access or engagement in relation to interventions among children and young people. The quality of the included papers was assessed, and data were extracted. Data were considered for meta-analysis, where possible.

**Results:**

A total of 3292 references were identified using search terms. Following the exclusion of duplicates and review by inclusion criteria, 40 studies were independently appraised for their methodological quality. A total of 16 studies were excluded owing to their low assessed quality and flawed critical elements in the study design. The studies focused on a variety of health conditions; type 1 diabetes, weight management and obesity, mental health issues, and sexual health were the predominant conditions. Most studies were conducted in developed countries, with most of them being conducted in the United States. Two studies reported data related to access and considered ethnicity and social determinants. No studies used strategies to enhance or increase access. All studies included in the review reported on at least 1 aspect of engagement. Engagement with interventions was measured in relation to frequency of engagement, with no reference to the concept of effective engagement.

**Conclusions:**

Most digital health interventions do not consider the factors that can affect access and engagement. Of those studies that measured either access or engagement or both, few sought to implement strategies to improve access or engagement to address potential disparities between groups. Although the literature to date provides some insight into access and engagement and how these are addressed in digital health interventions, there are major limitations in understanding how both can be enhanced to promote equity. Consideration of both access and engagement is vital to ensure that children and young people have the ability to participate in studies.

**Trial Registration:**

PROSPERO CRD42020170874; https://www.crd.york.ac.uk/prospero/display_record.php?RecordID=170874

## Introduction

### Background

Worldwide, access to many public services including health information and service provision is available through digital platforms [1]. The COVID-19 pandemic has accelerated the digital shift and highlighted the value it can bring to enabling access to health services and enhancing social connectedness [2]. However, equitable distribution of resources crucial for engaging with digital platforms—such as access to equipment, financial support for connectivity, and digital literacy—is uneven among populations. Consequently, certain groups have greater access to digital services than others [3,4]. It is crucial to focus on equity concerning access to digital health services, ensuring that the gap between those who can and cannot access these services is not widened further [5].

A plethora of literature exists on equity in health and health care; however, the key principles remain the same: that there should be equal access to health care for those in equal need of health care; equal use of health care for those in equal need of health care; and equal (equitable) health outcomes, for example, quality-adjusted life expectancy [6,7]. Equal access for equal need requires horizontal equity, conditions whereby those with equal needs have equal *opportunities* to access health care [8].

Health care providers are increasingly using digital technologies such as smartphones, websites, or SMS text messaging to communicate information to address health needs and in the delivery of health interventions [9]. Digital health interventions are programs that provide information and support for physical and mental health using digital technologies [10,11]. These interventions can be automated, interactive, and personalized, using user input or sensor data to shape feedback, treatment decisions, and treatment delivery [12].

Digital health interventions for children are increasing because of rapid technological advancements and the increasing interest of children and young people in technology [13]. Digital health interventions have been proposed to create opportunity to increase access to health care [14-16]. However, unless access to health care is equitable so that children and young people as consumers of health care within wider communities can use appropriate services in proportion to their need, inequities will create a divide in outcomes [17,18].

Although there is evidence for the effectiveness of digital health interventions developed for children and young people [19,20], understanding how issues related to access and variations by individuals, families, and communities are areas that have not been reviewed and require further discussion.

### Objective

This review aimed to identify the reports of access to, and engagement with, digital health interventions among children and young people. The review includes a report of data on access and engagement in studies that report on the effectiveness of digital health interventions as well as evaluations of strategies to increase access and engagement.

## Methods

The review followed the Joanna Briggs Institute (JBI) methodology for systematic reviews [21] in design and was conducted according to the PROSPERO protocol (CRD42020170874). The review was conducted in accordance with the PRISMA (Preferred Reporting Items for Systematic Reviews and Meta-Analyses) statement.

### Search Strategy

A scoping search was conducted to identify key papers and search terms to inform the search strategy. This included the key terms and medical subject headings engagement or equity of access or access to health care and digital health or mobile health or electronic health.

The search strategy was reviewed and refined by a research librarian. The base search strategy was developed on CINAHL. A total of 4 web-based databases, including CINAHL, MEDLINE, PsycINFO, and Embase, were searched for English language publications between January 2010 and August 2021 and updated in August 2022. A manual search in Google Scholar was also conducted. Gray literature sources including OpenGrey, ProQuest Dissertation and Theses (ProQuest), and Google and Google Scholar were also searched to identify unpublished studies. Multimedia Appendix 1 provides the full search strategy. EndNote (Clarivate) was used to remove duplicate citations before screening.

### Inclusion and Exclusion Criteria

The review included studies that reported data on access or engagement when reporting the effectiveness of digital health interventions for children and young people. The participants included school-aged children and young people aged 5-18 years. Parents or caregivers of children receiving health services were also included; however, studies that only reported the parent experience were excluded. Studies reporting on health interventions involving 1-way and 2-way communication including web-based platforms, mobile apps, videoconferencing, and SMS text messaging on access or engagement outcomes were included. Qualitative and quantitative studies were included in this review.

Studies that included children aged ≤4 years and ≥19 years were excluded. Studies that reported health professionals, such as nursing staff, medical personnel, health care management and administrators, or researchers, as the primary users of the digital health intervention were excluded. Studies reporting a telephone-based intervention with no additional technological function or where the intervention focused on health records such as patient portals or personal health records were excluded.

### Screening

The titles, abstracts, and full papers of the selected records were screened independently by 2 reviewers (SR and MJ) using the abovementioned inclusion and exclusion criteria. Any discrepancies were discussed, and disagreements were resolved by a third reviewer (LW). The reference lists of all included studies were reviewed to identify relevant papers that were not found in the electronic search.

### Assessment of Methodological Quality

The quality of the screened papers was critically appraised independently by reviewers (SR and LW) using the appropriate standardized critical appraisal instruments from JBI, including the Checklist for Randomized Controlled Trials, Checklist for Quasi-Experimental Studies, Checklist for Cohort Studies, Checklist for Analytical Cross Sectional Studies, and the Checklist for Qualitative Research [21].

### Data Extraction

Data were extracted from the included studies using an adapted version of the standardized data extraction tool from JBI [22]. Two reviewers (SR and MJ) extracted the data from the included papers, and a third reviewer (LW) verified the accuracy of the extracted data, with any disagreement resolved through discussion.

The extracted data included specific details about the study setting and context; the aim and objectives of the study; study design; the sampling of participants, sample size, and the characteristics of the study sample; and details about the interventions and engagement and access outcomes. All data were extracted following a thorough reading of the text to identify qualitative or quantitative findings relevant to the objectives and questions for the review. A second reviewer checked all the data extracted from each paper to enhance certainty.

### Data Synthesis

Owing to the heterogeneity between the studies on outcome measures, research design, and the intervention, a meta-analysis was not possible. The findings have been presented in narrative form including tables and figures to aid in data presentation. The process of data synthesis followed the JBI approach of meta-aggregation. The meta-aggregative approach is sensitive to the practicality and usability of the findings extracted and does not seek to reinterpret these findings. A strong feature of the meta-aggregative approach is that it enables the generation of statements in the form of recommendations that can guide researchers, practitioners, and policy makers. In this way, meta-aggregation contrasts with meta-ethnography or the critical interpretive approach to qualitative evidence synthesis, which focuses on reinterpretation and theory generation rather than aggregation.

## Results

### Study Inclusion

**Figure 1:**
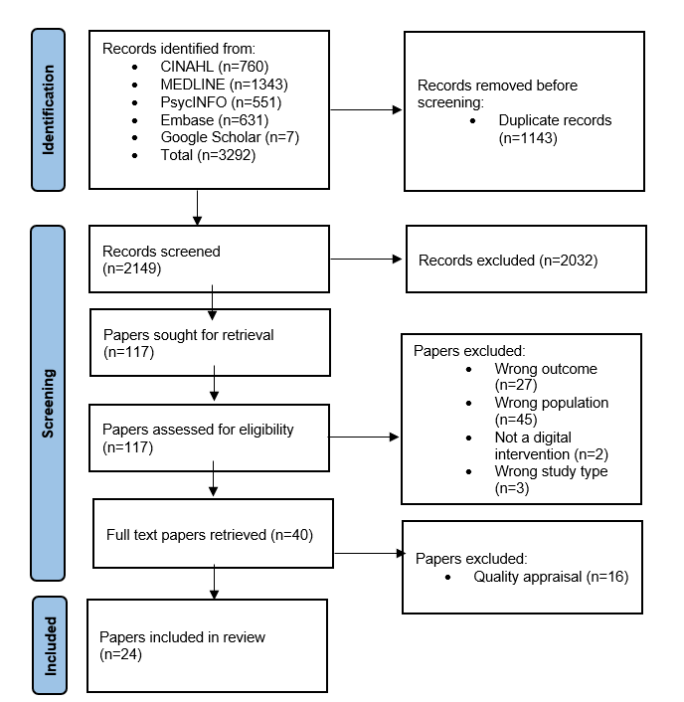
PRISMA (Preferred Reporting Items for Systematic Reviews and Meta-Analyses) flowchart of the study selection and inclusion process.

In total, 3292 references were identified using the search terms. The addition of secondary searches of reference lists and gray literature resulted in the identification of no further references. The exclusion of 1143 duplicates resulted in 2149 references. The titles and abstracts of the references were independently reviewed to determine if they met the inclusion criteria, and 2032 references were excluded. The remaining 117 references were retrieved in full text papers and reviewed by 3 reviewers (SR, MJ, and LW) using the inclusion criteria. A total of 77 studies were excluded as they did not meet the inclusion criteria. Of the 77 studies, 45 (58%) were excluded because the age of the child was outside the inclusion range, 27 (35%) did not report on access or engagement, 2 (3%) did not include a digital intervention, and 3 (4%) were opinion pieces or letters to the Editor. A total of 40 studies met the inclusion criteria (Figure 1).

### Methodological Quality

A total of 40 studies that met the inclusion criteria were independently appraised for their methodological quality. A total of 16 studies were excluded where the quality of the studies was assessed as low and critical elements of the study design were flawed (Tables 1-5). A cutoff was applied for each research design. A total of 5 randomized controlled trials (RCTs) were excluded because they were unclear or did not report on ≥6 items out of 13 items (Table 1). In addition, 7 quasi-experimental studies were excluded because they were unclear or did not report on ≥4 out of 9 (Table 2). All qualitative studies were retained (Table 3). The 1 cohort study was excluded because it did not meet 5 of the 11 items (Table 4). One cross-sectional study was excluded because it did not meet 4 of the 8 criteria (Table 5). Of note, the mixed methods study was assessed using the criteria for RCTs and qualitative studies for the relevant sections as per JBI guidance.

**Table 1:** Quality assessment. Randomized controlled trials.

Study	Randomization used for assignment of participants to treatment groups	Allocation to treatment groups concealed	Treatment groups similar at the baseline	Participants blind to treatment assignment	Those delivering treatment blind to treatment assignment	Outcomes assessors blind to treatment assignment	Treatment groups treated identically other than the intervention of interest	Follow-up complete and if not, were differences between groups adequately described and analyzed	Participants analyzed in the groups to which they were randomized	Were outcomes measured in the same way for treatment groups	Were outcomes measured in a reliable way	Was appropriate statistical analysis used	Was the trial design appropriate, and any deviations from the standard randomized controlled trial	Percentage of items assessed as met
Bergner et al [22], 2018	Yes	Yes	Yes	Unclear	Yes	No	Yes	Yes	Yes	Yes	Yes	Yes	Yes	85
Bunnell et al [23], 2017	Yes	Unclear	Unclear	Unclear	Unclear	No	Yes	Unclear	Yes	Yes	Yes	Yes	Unclear	46
Palermo et al [24], 2020	Yes	Unclear	Yes	No	Unclear	No	Yes	Unclear	Yes	Yes	Yes	Yes	Unclear	54
Hilliard et al [25], 2020	Yes	Unclear	Yes	Unclear	Unclear	Unclear	Yes	Yes	Yes	Yes	Yes	Yes	Yes	69
O’Connor et al [26], 2020	Yes	Unclear	Yes	No	No	No	Yes	No	Yes	Yes	Yes	Yes	Unclear	54
Palermo et al [24], 2020	Yes	Yes	Yes	No	Unclear	Yes	Unclear	Unclear	Yes	Yes	Yes	Yes	Yes	69
Perrino et al [27], 2018	Yes	Yes	Unclear	Unclear	Unclear	Unclear	Unclear	Unclear	Yes	Yes	Unclear	Yes	Unclear	38
Voss et al [28], 2019	Yes	No	Yes	No	Yes	Yes	Yes	Yes	Yes	Yes	Yes	Yes	Yes	85
Whittemore et al [29], 2013	Yes	Yes	Yes	Unclear	Yes	Yes	Yes	No	Yes	Yes	Yes	Yes	Yes	85
Widman et al [30], 2017	Yes	Yes	Yes	Yes	Unclear	Unclear	Yes	Yes	Yes	Yes	Yes	Yes	Yes	85
Ybarra et al [31], 2019	Yes	Yes	Yes	Yes	Yes	Unclear	Yes	No	Yes	Yes	No	Yes	Yes	77
Zhang et al [32], 2018	Yes	Unclear	Yes	No	Unclear	Unclear	Yes	Yes	Yes	Yes	Yes	Yes	Yes	69

**Table 2:** Quality assessment. Quasi-experimental studies.

Study	Clear what is the cause and what is the effect	Participants included in any comparisons similar	Participants included in any comparisons receiving similar treatment and care, other than the intervention of interest	There was a control group	Multiple measurements of the outcome both pre and post the intervention or exposure	Follow-up complete and if not, differences between groups in terms of their follow-up adequately described	Outcomes of participants included in any comparisons measured in the same way	Outcomes measured in a reliable way	Appropriate statistical analysis used	Percentage score
Anderson et al [33], 2018	Yes	Yes	Yes	No	Yes	Yes	Yes	Yes	Yes	89
Beaudry et al [34], 2019	Yes	Yes	Yes	No	Yes	Yes	Yes	Unclear	Yes	78
Brown et al [35], 2016	Yes	Yes	Yes	No	Yes	Yes	Yes	Unclear	Yes	78
Bunnell et al [23], 2017	Yes	Yes	Unclear	Yes	No	Yes	Yes	Yes	Yes	78
Fortier et al [36], 2016	Yes	N/A^a^	N/A	No	No	Unclear	Yes	Yes	Yes	44
Galy et al [37], 2019	Yes	Yes	Yes	No	Yes	Unclear	Yes	Yes	Yes	78
Kaushal et al [38], 2019	Yes	No	No	No	Unclear	No	N/A	Unclear	Yes	22
Kornman et al [39], 2020	Yes	N/A	No	No	Yes	No	N/A	Yes	Yes	44
Kosse et al [40], 2019	Yes	Yes	Yes	Yes	No	No	Yes	Yes	Yes	78
Larsen et al [41], 2018	Yes	Yes	Yes	No	Yes	Unclear	N/A	No	Yes	56
March et al [42], 2018	Yes	Yes	Yes	No	Yes	No	Yes	Yes	Yes	78
Myers et al [43], 2015	Yes	No	N/A	No	No	No	N/A	No	Yes	22
McGill et al [44], 2019	Yes	Yes	Yes	No	Yes	Yes	Yes	Yes	Yes	89
Padman et al [45], 2013	Yes	No	No	No	No	Yes	N/A	Yes	Yes	44
Pramana et al [46], 2014	Yes	No	No	No	No	No	No	Unclear	Yes	22
Sousa et al [47], 2015	Yes	Yes	Yes	Yes	Yes	Unclear	Yes	Yes	Yes	89
Tu et al [48], 2017	Yes	Yes	Yes	No	Yes	No	Yes	Unclear	Yes	67
Wingo et al [49], 2020	Yes	Yes	Yes	Yes	Yes	Yes	Yes	Unclear	Yes	78
Yen et al [50], 2019	Yes	Yes	Yes	No	Yes	Yes	Yes	Yes	Yes	89

^a^N/A: not applicable.

**Table 3:** Quality assessment. Qualitative studies.

Study	Congruity between the stated philosophical perspective and the research methodology	Congruity between the research methodology and the research question	Congruity between the research methodology and the methods used to collect data	Congruity between the research methodology and the representation and analysis of data	Congruity between the research methodology and the interpretation of results	Statement locating the researcher culturally or theoretically	Influence of the researcher on the research, and vice-versa, addressed	Participants and their voices adequately represented	Research ethical according to current criteria or, for recent studies	Conclusions drawn in the research report flow from the analysis and interpretation, of the data	Percentage score
Bergner et al [22], 2018	No	Yes	Yes	Yes	Yes	No	No	Yes	Yes	Yes	70
LeRouge et al [51], 2016	No	Yes	Yes	Yes	Yes	No	No	Yes	Yes	Yes	70
Lopez et al [52], 2020	Yes	Yes	Yes	Yes	Yes	No	No	Yes	Yes	Yes	80
Tolou-Shams et al [53], 2019	Unclear	Yes	Yes	Yes	Yes	No	No	Yes	Yes	Yes	70

**Table 4:** Quality assessment. Cohort study.

Study	Two groups similar and recruited from the same population	Exposures measured similarly to assign people to both exposed and unexposed groups	Exposure measured in a valid and reliable way	Confounding factors identified	Strategies to deal with confounding factors stated	Participants free of the outcome at the start of the study	Outcomes measured in a valid and reliable way	The follow-up time reported and sufficient to be long enough for outcomes to occur	Follow-up complete, and if not, were the reasons for loss to follow-up described and explored	Strategies to address incomplete follow-up used	Appropriate statistical analysis used	Percentage score
Cueto et al [54], 2019	Yes	No	No	No	Unclear	Yes	No	Yes	Yes	Yes	Yes	55

**Table 5:** Quality assessment. Analytical cross-sectional studies.

Study	Were the criteria for inclusion in the sample clearly defined?	Were the study subjects and the setting described in detail?	Was the exposure measured in a valid and reliable way?	Were objective, standard criteria used for measurement of the condition?	Were confounding factors identified?	Strategies to deal with confounding factors stated	Outcomes measured in a valid and reliable way	Appropriate statistical analysis used	Percentage score
Dowshen et al [55], 2015	Yes	Yes	Yes	No	No	No	No	Yes	50
Piatkowski et al [56], 2020	Yes	Yes	Yes	Yes	Yes	No	Yes	Yes	88

### Characteristics of the Studies

Of the 24 studies included in the review (Table 6), 7 (29%) used an RCT design, 12 (50%) were quasi-experimental studies, and 3 (13%) used a qualitative study design. One study used an analytical cross-sectional study design and 1 used a mixed methods design.

**Table 6:** Study characteristics.

Study	Health condition	Aim and objectives	Country	Study setting	Study design	Type of digital intervention	Age	Gender
Anderson et al [33], 2018	Sickle cell disease	To examine the feasibility of the Intensive Training Program (ITP), a mobile health intervention for youths with sickle cell disease to promote disease knowledge, adherence, and patient-provider communication.	United States	Pediatric sickle cell disease clinic	Quasi-experimental	Mobile app	Children: mean age of children 13 (SD 3.33) years	Children: 16 (50%) children were female
Beaudry et al [34], 2019	Children transitioning from pediatric to adult care with chronic illness	To test the feasibility of a texting platform aimed at increasing engagement among teenagers while teaching essential self-care skills while transitioning to adult focused care	United States	Pediatric inﬂammatory bowel disease, cardiology, and type 1 diabetes specialty clinics	Quasi-experimental	Text message	Children: mean age of children 15 years; 2 aged 14 years; 1 aged 15 years; 9 aged 16 years; and 1 aged 17 years	Children: sex of children not provided
Bergner et al [22], 2018	Type 1 diabetes	To evaluate the acceptability and feasibility of Check It! a positive psychology intervention to improve adherence in adolescents with T1D^a^	United States	Outpatient pediatric diabetes clinic	Mixed method (RCT^b^ and qualitative)	Text message	Children: mean age of adolescents 14.8 (SD 1.5) years	Children: 63 (52.5%) female participants and 57 (47.5%) male participants
Brown et al [35], 2016	Sexual health	To evaluate a behavior change intervention targeting sexual health service uptake among young people delivered using digital media	United Kingdom	Secondary schools	Quasi-experimental pretest posttest design	Website and mobile app	Children: mean age at baseline 15.7 (SD 1.51) years	Children: at baseline 158 (55%) female and 129 (45%) male participants; at follow-up 94 (41%) female 134 (59%) males
Bunnell et al [23], 2017	Mental health	To examine access and completion of a web-based disaster mental health intervention in adolescents and their caregivers affected by the spring 2011 tornadoes in Missouri and Alabama	United States	Community	Quasi-experimental; pretest posttest design	Website	Children: mean age of rural children was 14.5 (SD 1.76) years; mean age of urban children was 14.6 (SD 1.74) years; parents or caregivers: mean age of rural caregivers was 45.0 (SD 9.54) years; mean age of urban caregivers was 45.4 (SD 9.38) years	Children: 329 (49%) rural female participants and 347 (51%) rural male participants; 658 (50%) urban females and 663 (50%) urban males; parents or caregivers: 493 (72.9%) rural caregivers were female and 183 (27.1%) were male; 980 (74.2%) urban care givers were female and 341 (25.8%) were male
Galy et al [37], 2019	Overweight and obesity	To investigate a technology-based program combining education, objective measures of PA^c^), and self-assessment of goal achievement delivered to Pacific adolescents	New Caledonia	School	Quasi-experimental pilot study	Mobile app and wearable tracker device	Children: mean age of children 11.9 (SD 0.57) years; age ranged from 12 to 14 years	Children: sex not provided
Hilliard et al [25], 2020	T1D	To evaluate the feasibility and acceptability of a behavioral intervention delivered to parents of adolescents with T1D via mobile-friendly web app	United States	Diabetes clinic in the hospital	RCT	Mobile app	Children: mean age of children 15.3 (SD 1.5) years; parents: not provided	Children: 47 (59%) female participants and 33 (41%) male participants; parents: 64 (80%) female and 16 (20%) male
Kosse et al [40], 2019	Medication self-management asthma	To explore the use and the effective engagement of adolescents aged 12-18 years with the Adolescent Adherence Patient Tool	The Netherlands	Community	Quasi-experimental	Mobile app	Children: mean age of children 15.0 (SD 2.0) years	Children: 48 (55%) female participants and 39 (45%) male participants
LeRouge et al [51], 2016	Weight management (overweight)	To investigate the use of animated avatars and virtual agents to deliver computer-based interventions for chronic weight management ins adolescents	United States	Camp Jump Start	Qualitative	Virtual avatars	Children: mean age of adolescents not provided	Children: sex of children not provided
Lopez et al [52], 2020	Substance use and HIV	To evaluate a technology-based approach to delivering culturally tailored, integrated substance use disorder and HIV risk behavior prevention programs to African American female youths	United States	School and community	Qualitative	Telemedicine	Children: age ranged from 13 to 18 years	Children: all (100%) female participants
March et al [42], 2018	Mental health (anxiety)	To examine program adherence, satisfaction, and changes in anxiety with a publicly available online, self-help iCBT^d^ program (BRAVE Self-Help)	Australia	Community	Quasi-experimental	Website	Children: mean age of children 12.9 (SD 2.97) years	Children: 2938 (66.4%) female participants and 1406 (31.8%) male participants; 81 (1.8%) participants identified as another gender category
McGill et al [44], 2019	Diabetes type 1	To evaluate an SMS text messaging intervention in teenagers with T1D assessing factors associated with text responsiveness and glycemic benefit	United States	Outpatient clinic	Quasi-experimental	Text message	Children: mean age of children 14.9 (SD 1.3) years	Children: 76 (52%) female participants and 70 (48%) male participants
Palermo et al [24], 2020	Chronic pain	To evaluate effectiveness and implementation of a digital health delivered psychological intervention for children aged 10-17 years with chronic pain	United States	Pain clinics	Stepped-wedge cluster randomized trial	Mobile app	Children: mean age of children 14.5 (SD 1.9) years	Children: 117 (81.8%) female participants and 26 (19.2%) male participants
Piatkowski et al [56], 2020	Obesity	To examine user characteristics and parenting practices associated with adolescents’ initial use of the Aim2Be app; a health behavior modiﬁcation intervention	Canada	Community	Analytical cross-sectional study	Mobile app	Children: mean age of children 14.9 (SD 1.5) years	Children: 184 (49.6%) female participants and 187 (50.4%) male participants
Sousa et al [47], 2015	Overweight and obesity	To evaluate the effectiveness of an e-therapeutic platform (Next.Step), aiming to promote weight management skills and the adoption of health-promoting lifestyles	Portugal	Pediatric obesity clinic	Quasi-experimental	Website	Children: mean age of children 14.2 (SD 1.51) years	Children: 48 (51.1%) female participants and 46 (48.9%) male participants
Tolou-Shams et al [53], 2019	Mental health and substance abuse	To examine the acceptability of a dyadic (youth and caregiver) SMS text messaging intervention to enhance treatment engagement of the youths attending face-to-face community-based treatment, as referred by probation staff	United States	Community-based Juvenile Probation Department and community-based provider organization	Qualitative	Text message	Children: mean age of children was 17.0 years; caregiver: age ranged from 35 to ≥65 years.	Children: 6 (75%) female participants and 2 (25%) male participants; caregiver: 4 (80%) female and 1 (20%) male
Tu et al [48], 2017	Overweight and obesity	To determine whether adolescent and parental adherence to components of an e-health intervention resulted in change in adolescent BMI and waist circumference (WC) z-scores in a sample of overweight/obese adolescents	Canada	Children’s Hospital Endocrinology and Diabetes Clinic and Center for Healthy Weights program in British Columbia and by other sources	Quasi-experimental	Website	Children: mean age of children 13.2 (SD 1.8) years; parents: mean age of parents 45.8 (SD 6.2) years	Children: 91 (57.2%) female participants and 68 (42.8%) male participants; parents: 135 (84.9%) female participants and 24 (15.1%) male participants
Voss et al [28], 2019	Autism	To evaluate the efficacy of Superpower Glass, an artificial intelligence–driven wearable behavioral intervention for improving social outcomes of children with ASD^e^	United States	Home environment	RCT	Wearable glasses	Children: mean age of 8.4 (SD 2.46) years	Children: 8 (11%) female participants and 63 (89%) male participants
Whittemore et al [29], 2013	Type 1 diabetes	To compare the demographic and clinical characteristics of young people with T1D on recruitment, participation, and satisfaction with eHealth programs	United States	Clinical sites	RCT	Website	Children: mean age of 8.4 (SD 2.46) years	Children: 177 (55.3%) female participants and 143 (44.7%) male participants
Widman et al [30], 2017	Sexual health	To assess the feasibility and acceptability of Project HEART providing sex education focusing sexual communication skills to reduce the risk of HIV/STDs^f^ and unplanned pregnancy among youths	United States	High schools	RCT	Website	Children: mean age of 12.3 (SD 1.1) years	Children: 107 (100%) female participants
Wingo et al [49], 2020	Children with physical disabilities	To test the usability and preliminary efﬁcacy of an eHealth and telecoaching intervention compared with telecoaching alone	United States	Pediatric rehabilitation medicine clinics	Quasi-experimental	Website	Children: mean age of 11.3 (SD 3.3) years; parents: mean age of parents not provided	Children: 29 (58%) female participants and 21 (42%) male participants; parents: 45 (90%) female participants and 5 (10%) male participants
Ybarra et al [31], 2019	HIV prevention	To determine whether technology is an appropriate delivery mechanism for adolescent-focused HIV preventive programing in South Africa	South Africa	Schools	RCT	Text message	Children: mean age of 17.5 (SD 1.2) years	Children: 647 (63.7%) female participants and 368 (36.3%) male participants
Yen et al [50], 2019	Mental Health (suicidal behavior)	To examine feasibility, acceptability, and clinical outcomes of a positive affect skills–based technology-assisted program in an acute setting	United States	Adolescent inpatient psychiatric unit	Quasi-experimental	Text message	Children: mean age of 15.9 (SD 1.5) years	Children: 15 (75%) female participants and 5 (25%) male participants
Zhang et al [32], 2018	Diabetes type 1	To investigate adolescents with T1D engagement with an SMS text messaging intervention	United States	Diabetes clinic	Randomized pilot study	Text message	Children: mean age of 15.0 (SD 1.3) years	Children: 25 (52.1%) female participants and 23 (47.9%) male participants

^a^T1D: type 1 diabetes.

^b^RCT: randomized controlled trial.

^c^PA: physical activity.

^d^iCBT: internet-based cognitive behavioral therapy.

^e^ASD: autism spectrum disorder.

^f^STD: sexually transmitted disease.

The studies focused on a variety of health conditions; type 1 diabetes (4/24, 17%), weight management and obesity (5/24, 21%), mental health issues (4/24, 17%), and sexual health (3/24, 13%) were the predominant conditions (Table 6). Most studies (23/24, 96%) were conducted in developed countries. Most studies (15/24, 63%) were conducted in the United States.

Of the 24 studies included in the review, 10 (42%) recruited participants from outpatient clinics, 1 (4%) recruited from the hospital setting, 4 (17%) recruited in schools, and 8 (33%) within community settings. One study recruited participants from both a school and a community setting.

In more than half of the studies (16/24, 67%), more females were recruited than males. In 3 studies, the gender of the child was not provided [23,33,51].

### Type of Digital Interventions

Overall, 38% (9/24) of the digital health interventions were web based, 21% (5/24) of the interventions were mobile apps, 29% (7/24) of the interventions used SMS text messaging, 4% (1/24) of the interventions used a website and a mobile app, 4% (1/24) of the interventions were a telemedicine intervention with participants logging in on their home computer or tablet, and 8% (2/24) of the digital interventions combined a website and digital wearable glasses and an app and wearable tracker (Table 6).

### Access and Engagement

#### Access to Digital Health Interventions

The 2 studies that reported access and digital health interventions included 1 that reported on access related to race and ethnicity and access by income and 1 that reported on gender differences in accessing services (Table 7).

**Table 7:** Report of access and engagement.

Study	Number of participants enrolled	Intervention period	Data reported on access	Engagement; logged in; or interacted at least once	Engagement; frequency; average per day or week	Engagement; intensity of engagement	Engagement; completion of the course	Engagement; acceptance satisfaction
Anderson et al [33], 2018	32 children completed the baseline survey	90 days (6 weeks) participants to enter medication daily	No data reported	28 (87%) participants logged in	Participants logged in average 18 of the 30 days (60% of participants logged in each day)	37% tracking daily entry	27 (84%) participants completed track an entry of medication each day	Ranged from 41.7% to 91.7%
Beaudry et al [34], 2019	13 children enrolled	24 weeks—weekly text messages sent	No data reported	13 (100%) children responded to the chatbot	97% responded to weekly text message	Responses rates ranged from 85% to 100% response to the text message each week	13 children, 100% responded to the last text of the study period. 12 (92%) children completed the final survey	Satisfaction was not measured on the survey. Children reported being motivated to respond to the texts because of its “ease of use” and because they were “friendly.”
Bergner et al [22], 2018	120 parent child dyads enrolled	8 weeks; intervention group to answer weekly text message	No data reported	Information not provided	14% teenagers answered weekly phone reminders (control group) vs 67% in the text (intervention) group (*t*=7.97; *P*<.001)	No other measurement provided	89% of the adolescents and 92% of the parents completed the 3-month follow-up survey	Adolescents and their parents were satisfied with the study, with >87% noting a positive experience.
Brown et al [35], 2016	287 children enrolled at baseline	6 weeks	A digital intervention approach had a signiﬁcant positive effect on psychological barriers to and antecedents of service access among females. Males reported greater conﬁdence in service access than females.	100%	No measured	At follow-up, all participants reported having accessed the website or web app at least once. 45% had visited ≥2 main intervention pages. 36% indicated that they had not visited any of the core website pages and 21% indicated that they had visited only one of the 19 main intervention pages.	Not measured	Not measured
Bunnell et al [23], 2017	2000 families (parent child dyad)	Intervention period not provided	No data reported	485 (36.7%) urban adolescents and 223 (33.0%) rural adolescents accessed the resource. 503 (38.1%) urban caregivers and 233 (34.5%) rural caregivers accessed the resource.	Not measured	Not measured	384 (79.2%) urban adolescents and 170 (76.2%) rural adolescents completed the course. 313 (62.2%) urban and 128 (54.9%) rural caregivers completed the course.	Not measured
Galy et al [37], 2019	24 adolescents	4 weeks to 8 one-hour modules	No data reported	24 (100%) adolescents used the electronic tracking device	24 (100%) adolescents wore the electronic tracking device daily	Not measured	21 (84%) adolescents competed the program.	95% of the adolescents rated their satisfaction with the modules as “fun.”
Hilliard et al [25], 2020	80 families enrolled. At baseline randomized to 55 family’s intervention and 25 families usual care control	3 to 4 months	No data reported	All 55 (100%) intervention arm families (parents) downloaded the app and logged in at least one time	53 participants (parents; 96%) logged in at least 1 additional time. 91% of parents used the app ±2 days per week on average. 79.9% of parents logged in each day.	96% of the participants used the strengths tracking section of the app. 90% of the participants viewed the strengths summaries.	78 families (98%) completed follow-up	Intervention participant responses (n=50) on the USE^a^. questionnaire indicated high acceptability of the intervention. Feedback from 48 parents was positive.
Lopez et al [52], 2020	58 African American adolescents	S 11 weekly; 1-hour group sessions with youth participants and 1 20-minute individual session with each parent of participants at some point between weeks 5 and 9 (totaling 12 weeks)	No data reported	53 (91%) adolescents completed the baseline	—^b^	—	39 (67%) completed the intervention	100% would recommend the program to a friend
Kosse et al [40], 2019	103 patients enrolled	6 months	No data reported	87 (84%) patients logged in to the app. 16% of the patients did not download the app.	86 adolescents used the app 1975 times between October 2015 and April 2017. The median app use per person was 17 times.	51% watched at least 1 movie. 65 (75%) adolescents sent or received ≥3 chat messages. 18 adolescents used the peer chat.	26 (weekly) reminders sent to complete the app—individually completed the app 10 times.	Not measured
LeRouge et al [51], 2016	70 adolescents	Intervention period not provided	A structured protocol of questions including general background questions (ie, age, technology access questions, level of avatar, or virtual agent experience) and then reviewed midfidelity mock-ups of 7 types of graphical embodiments of the character, for the virtual self-avatar or virtual agent.	70 (100%)	Not measured	Not measured	Not measured	Not measured
March et al [42], 2018	4425 young people enrolled	20 weeks with 10 sessions	No data reported	3467 (78.4%) completed the first session	Not measured	48.05% (2126/4425) of the registered participants completed only 1 or 2 sessions. 24.75% (1095/4425) of the participants completed at least 3 sessions.	3.6% (163/4425) completed all 10 sessions	The mean total satisfaction rating was 17.72 (SD 5.16) out of a maximum 25
McGill et al [44], 2019	151 young people enrolled	18 months	No data reported	147 (97%) young people received the SMS text messaging intervention. Received a daily text message to check blood glucose levels.	Over 18 months, 49% of young people responded with ≥1 blood glucose result on ≥50% of days. Declined over time (0 to 6 months 60% response—7 to 12 months 50% daily response); 13 to 18 months 43% daily response	Not measured	Not measured	Not measured
Palermo et al [24], 2020	143 youths enrolled: 73 youths assigned to the treatment group and 70 youths to the control group	8 weeks	No data reported	68 (97%) youths downloaded the app and 54 youths (74%) completed at least 1 module of the intervention.	Not measured	Youths completed an average of 3.1 modules; range 5 (0 to 8)	20 (27%) youths completed the intervention program.	85.7% of youths and rated the WebMAP program as moderately to highly acceptable on the Treatment Evaluation Inventory
Piatkowski et al [56], 2020	371 adolescents and parent dyads enrolled and completed the baseline assessment	Not provided	No data reported	294 (79.2%) adolescents used the app	Not measured	Not measured	Not measured	Not measured
Sousa et al [47], 2015	94 adolescents enrolled (48 adolescents enrolled in the experimental group and 46 adolescents enrolled in the control group)	24 weeks	No data reported	25 (52.1%) adolescents in the experimental group logged in to the website.	On average, accessed the platform 10.68 times (SD 18.92)	On average analyzed 7.9 (SD 9.25) resources and read 31.8 (SD 47.56) messages from the forums during the 24-week period.	13.7% of the adolescents in the experimental group completed the activities.	Satisfaction was not measured.
Tolou-Shams et al [53], 2019	8 youths	6 months	No data reported	Not measured	Not measured	Not measured	7 (87.5%)	Not measured
Tu et al [48], 2017	159 (90%) adolescent parent dyads participated	8 months	No data reported	15 (9.4%) adolescents and 50 parents (31.5%) did not log in to the intervention website during the entire study period.	Over the 33-weeks intervention adolescents logged into the website an average of 13.4 weeks, and parents logged into the website an average of 7.5 weeks	Adolescents mean percentage of web pages viewed per week, where a total of 83 and 78 pages could be viewed in the first and last 4 months, respectively (typically there were 4-5 pages per week to view).	On average, adolescents and parents completed 28% of the web pages viewed.	Satisfaction was not measured
Voss et al [28], 2019	71 families enrolled; 40 (56.3%) were randomly assigned to the treatment and 31 (43.7%) to the control group	6 weeks; 20-minute sessions at home 4 times a weeks	No data reported	27 (67.5%) of the 40 treatment families engaged with the Superpower glasses.	Families used the glasses 12.1 times over the 6 weeks.	27 (67.5%) families used each of the 3 engagement activities at least once, used the device at home for 20 min 3 times per week. Participants played guess the emotion in 39.8%, capture the smile 23.8%, and unstructured free play 36.4%.	24 (60%) families completed the intervention	Satisfaction was not measured
Whittemore et al [29], 2013	320 youths enrolled: 167 were allocated to TeenCope intervention and 153 were allocated to managing diabetes intervention.	5 sessions	Black, Hispanic, or mixed-race and -ethnicity youths with type 1 diabetes were less likely to enroll in digital health interventions than White and higher-income youths	148 (90.3%) youths who received the intervention logged in	Not measured	Not measured	250 (78.1%) youths completed at least 4 of 5 sessions. The mean number of sessions completed was 4.08 (SD 1.64) across both groups. 39 (12.2%) completing 1 to 3 sessions, and 31 (9.7%) completing no sessions.	Satisfaction was high with mean satisfaction score was 3.97 (SD 0.71) for TEENCOPE (1 is not at all satisfied and 5 is very satisfied)
Widman et al [30], 2017	107 participants randomly assigned to the intervention group and 115 participants assigned to the control group.	1 session; 45 minutes to complete	No data reported	107 (100%) participants interacted with the website	Not measured	Not measured	107 (100%) participants completed the intervention	Participants found the program to be highly acceptable with 79% of participants reported they would come back to the website again, 88% would recommend the program to a friend, and 94% plan to use the information they learned in the future
Wingo et al [49], 2020	65 parent and child dyads consented and randomized and a total of 32 dyads randomized to the eHealth group and 33 to the telephone only group.	12 weeks	No data reported	24 (75%) eHealth group received the intervention; 26 (78.7%) telephone only group received the intervention.	Not measured	Mean days journal entry: 45.6 food, 46.1 water, and 42.1 physical activity	17 (67%) in the eHealth group compared with 23 (92%) of telephone only group completed the intervention.	Parents indicated they valued phone calls more than the eHealth platform
Ybarra et al [31], 2019	303 youths; 150 intervention and 153 control	8-10 daily text messages sent over 5-week period	No data reported	98% of the intervention participants sent or received a text message	Not measured	Not measured	Not measured	93% of the intervention participant said they somewhat or strongly agreed that they liked the program
Yen et al [50], 2019	20 (83%) adolescents enrolled	4 weeks	No data reported	100% responded	On average, participants responded to text prompts on 72.4% of days	Not measured	19 adolescents completed the intervention.	The intervention was described as good or excellent by >90% of the parents and 100% of the adolescents
Zhang et al [32], 2018	48 adolescents were enrolled. 24 adolescents and their caregivers in intervention group and 24 in the education group.	8 weeks	No data reported	87% responded	The mean response rate was 76 to the 4 to 5 text messages per week overall. Responses waned over the 8-week period, from 87% in week 1 to 81% in week 5 and 62% in week 8.	Not measured	Not measured	Not measured

^a^USE: Usefulness, Satisfaction, and Ease of use.

^b^Data not reported.

#### Race and Ethnicity

Equity of service use based on race and ethnicity was explored in 1 study. Whittemore et al [29] reported that Black, Hispanic, or mixed-race youths with type 1 diabetes were less likely to enroll in digital health interventions than White and high-income youths. However, once enrolled, youths of diverse races and ethnicities with type 1 diabetes were as highly satisfied with the eHealth programs as White youths. The results suggest that eHealth programs have the potential to reach diverse youth groups and to be relevant to them; however, considerations relating to access need to be addressed in the study design.

One study reported on access related to gender. Brown et al [35] reported that the digital intervention had a signiﬁcant positive effect on psychological barriers to and antecedents of service access among females. Males reported greater conﬁdence in service access than females and signiﬁcantly increased service access by the second follow-up.

Equity of service use based on income was explored in 1 study. Whittemore et al [29] reported that low-income youths were less likely to participate, possibly because of access. However, once enrolled, youths of diverse races and ethnicities and low-income youth with type 1 diabetes were as highly satisfied with the eHealth programs as White youths and those with higher income.

### Engagement With Digital Interventions

#### Overview

Engagement with the digital health intervention was measured by the frequency and intensity of engagement, satisfaction with the digital health intervention, and changes in knowledge or behavior. Of the studies that reported on engagement, most used system use data to capture how the intervention was used by each participant. The studies reported on various aspects of use data including initial log-in, frequency, intensity, and duration of engagement with the program, as described in Table 7.

#### Initial Log-In

Once enrolled in a digital health intervention, most participants logged in and engaged with the intervention. The percentage of enrolled participants logging in at least once to the digital intervention ranged from 35.6% [23] to 100% [30,34,35,37,50]. One study did not provide this information [22]. In 16 studies, more than three-quarters of the participants logged on at least once to the digital intervention (Table 7).

#### Frequency of Engagement

Frequency of engagement was measured by the log-in data, number of log-ins recorded per participant, average log-ins per unit of time or total for intervention duration, visits to the site, number of visits per participant, average per unit of time, or total time of visits. Overall, 42% (10/24) of the studies reported the average number of log-ins per unit of time. The measurement of frequency varied across the studies with either daily or weekly measurement with the unit of measurement dependent on the study aims and the frequency of the delivery of the intervention.

Overall, 21% (5/24) of the studies reported on engagement on a daily basis with between 49% [44] to 100% [37] of the participants engaging daily with the intervention. Moreover, 29% (7/24) of the studies reported weekly engagement with the digital health intervention, 13% (3/24) of the studies reported the percentage of participants engaging weekly, and 17% (4/24) of the studies reported the average weekly engagement with the website or app.

The most frequent measurement of the frequency of engagement was daily or weekly response to text messages by participants as reported in 6 studies.

Zhang et al [32] found that adolescent sex was signiﬁcantly related to engagement (*t*=2.42; *P*=.02), with boys demonstrating higher response rates (88%) than girls (67%). However, Whittemore et al [29] found no significant gender difference in enrollment and participation in an eHealth program for adolescents with type 1 diabetes.

#### Intensity of Engagement and Type of Behavior

The intensity of engagement was measured by pages viewed, modules viewed, number of emails sent, number of posts, and number of experts accessed. Three studies measured the number of log-ins per participant and reported the number of times an app or web page was visited. Zhang et al [32] reported that race and ethnicity were signiﬁcantly related to engagement (*t*=3.48; *P*=.04), with White, non-Hispanic youths responding to more messages (80%) than youths in racial and ethnic minority groups (45%).

One study measured functions used stating the number and percentage of participants who used the 5 functions within the intervention platform [40].

#### Completion of Modules and Courses

Most studies measured either completion of modules or completion of the course, with completion rates ranging from 3.6% to 100%, with most studies reporting >80% of participants completing modules or the course. Completion of modules, web pages, and courses were measured in 16 studies. In the study with the lowest completion rate [42], completion of all 10 sessions was low (3.6%), but 48% of the participants completed some sessions [40]. Although completion rates were reported in 16 studies, understanding whether these were higher or lower than expected or in direct comparison to face-to-face or other nondigital intervention approach was not clear. Completion of the intervention sessions was high in several studies (Table 7); for example, 84% of the participants completed the intervention in 2 studies [33,37], 95% of the participants completed the intervention in another study [50], to 100% of the participants completing the intervention [37]. The results did not provide insight into whether the digital nature of the intervention increased, decreased, or had a neutral impact on completion rates.

#### Satisfaction

Satisfaction was measured in 14 studies, with satisfaction measurement methods varying across the studies (Table 7). Of the 14 studies that assessed satisfaction, participants were generally satisfied with the digital intervention, and in 1 study [49] participants were more satisfied with telephone calls than the digital alternative. When reported, satisfaction rates were high, ranging from 42% [33] to 93% [31].

## Discussion

### Principal Findings

This review found that few studies have reported on how they addressed access and engagement of children and young people in digital health interventions. Most studies (23/24, 96%) included in the review were conducted in developed countries, mainly the United States. Only 2 studies reported data related to access, and no study reported the use of strategies to enhance or increase access. All studies included in the review reported on at least 1 aspect of the engagement of children and young people in interventions. Engagement was assessed in relation to frequency but did not consider whether the level of engagement achieved could be considered effective.

Access to health care includes both the availability of services and the ability of individuals and populations to access services. Inequities in access to health care tend to affect the most susceptible people in our communities and those with the most complex health care needs [17,57]. Until now, the examination of young people's access to digital health interventions has primarily focused on reviewing their engagement after enrollment in the study. However, there has been minimal consideration of equity issues regarding access before enrollment or engagement after enrollment among different groups. There is much work to be done in carefully mapping the factors that may affect access within a population during the conception of a study and planning for how to improve equity in relation to access before recruitment begins. The World Health Organization [58] has developed a framework for planning, developing, and implementing youth-centered digital health interventions. The framework provides guidance on the key considerations at each stage, including whether a digital solution is the best approach and consulting with young people. Examples of considerations for researchers and others to deliberate include ownership of, and access to, digital devices; connectivity in a geographical area; and community consultation to understand the cultural, social, family, and individual beliefs and behaviors related to technology, health, and behavioral change to create a user-centered designed intervention.

Variability in the measurement of engagement with digital health interventions reflects the diversity, complexity, and multiple aims of the digital health interventions. Although there is variability in the measurement of engagement, most young people in the studies included in this review engaged with the digital health interventions once enrolled. The measurement of engagement with interventions was based on use data, frequency and intensity of engagement, and user satisfaction data. There has been no exploration of the relationship between engagement with the digital intervention and the outcome measures. The concept of “effective engagement” [19] was not explored in the papers included in the review. The concept of promoting effective engagement rather than simply more engagement is an area that could yield valuable insights into how to support young people to achieve the goals and intended outcomes of a digital health intervention. Exploring and recognizing the combination of measures to promote and support “effective engagement” is an area for development with the potential to test multidimensional models of engagement [1,59].

The digitalization of health has the potential to improve health outcomes by empowering young people to become active custodians of their own health. There is the potential to improve access and health outcomes for traditionally underserved groups where smartphone ownership and use are higher than the general population [60,61]. However, caution has been advised regarding the digitalization of health, as it tends to favor certain groups while potentially having negative impacts on others. Although there has been exponential growth in the use of the internet, access to health information remains unequal [61].

Equal use for equal need requires conditions whereby those who have an equal need for health care make equal use of health care. Compared with equal access for equal need, this equity principle requires more proactive efforts. Areas related to fiscal and social policy, that influence education, housing conditions, and nutrition, are highly influential and speak to fundamental determinants of health. To promote access and engagement, researchers must first recognize the importance and value of considering these factors and preempt, plan, and document their efforts to make progress.

The limitations of this review include the search for, and inclusion of, papers published in English only. The heterogeneity of the papers meant that a meta-analysis was not possible and a narrative summary was completed. The review included studies that reported on either access or engagement or both; however, improving or addressing these concepts was not the primary aim of the studies. Where the 2 concepts are fundamental to the design and effectiveness of digital interventions, a strength of the review lies in the inclusion of all studies that report on the consideration of access and engagement.

### Conclusions

The review identified several gaps and raised important questions for further investigation. Most of the studies reporting on access or engagement, did not seek to improve access to digital technology and focused on the frequency of engagement. Future work should explore how access and engagement can be considered preemptively and assessed throughout the intervention, with the goal of improving the equity of access and effective engagement with digital interventions.

## Appendix

Multimedia Appendix 1 Search strategy.

Multimedia Appendix 2 PRISMA checklist.

## Abbreviations

JBI: Joanna Briggs Institute
PRISMA: Preferred Reporting Items for Systematic Reviews and Meta-Analyses
RCT: randomized controlled trial

## Data Availability

Data are presented in the manuscript and Multimedia Appendix 1.
