# Supplementary material for: The Report of Access and Engagement With Digital Health Interventions Among Children and Young People: Systematic Review
Source: JMIR Pediatr Parent. 2024 Jan 17;7:e44199. doi: 10.2196/44199 (PMC10831666; doi:10.2196/44199)
Supplement: Multimedia Appendix 1 [file pediatrics_v7i1e44199_app1.docx]

**Appendix 1: Search strategy**

CINAHL, Medline, PsychInfo and Embase searched on 6 July 2020.

| Search | Query |
| --- | --- |
| #1 | (MH "Child") OR "young people" OR "young person" OR (MH "Young Adult") OR "teen*" OR (MH "Adolescent") OR "child” OR "young adult" OR "adolescen*" |
| #2 | AND |
| #3 | AB equit* OR "engag*" "health equity" OR "equity of access” OR "access to healthcare" OR AB "access" |
| #4 | AND |
| #5 | "digital health" OR "ehealth" OR"e-health" OR "digital health care" OR "mobile health" OR "digital medicine" OR "digital technology" OR "electronic health" OR "telehealth" OR "mhealth" OR (MH "Smartphone") OR "telecare" OR "m-health" OR "telemedicine" OR (MH "Telemedicine") OR cellphone*" OR "smartphone*" OR "remote consultation" OR "internet based intervention" OR "web-based intervention" OR "electronic health intervention" OR (MH "Mobile Applications") OR (MH "Cell Phone Use") OR (MH "Remote Consultation") OR "health information technology" OR (MH "Internet-Based Intervention") OR (MH "Telehealth") |
|  | AND |
|  | "strategy” OR "approach" OR "trial" OR "program" OR "intervention" |
| LIMITED | Published 2010 onwards; English Language |
